# Co-operative inhibitory effects of hydrogen peroxide and iodine against bacterial and yeast species

**DOI:** 10.1186/1756-0500-6-272

**Published:** 2013-07-15

**Authors:** Elena I Zubko, Mikhajlo K Zubko

**Affiliations:** 1 School of Healthcare Science, Manchester Metropolitan University, John Dalton Building, Chester St, Manchester M1 5GD, UK

**Keywords:** Antimicrobials, Synergism, Iodine, Hydrogen peroxide, Escherichia, Pseudomonas, Staphylococcus, Saccharomyces, Candida, Genotoxicity

## Abstract

**Background:**

Hydrogen peroxide and iodine are powerful antimicrobials widely used as antiseptics and disinfectants. Their antimicrobial properties are known to be enhanced by combining them with other compounds. We studied co-operative inhibitory activities (synergism, additive effects and modes of growth inhibition) of hydrogen peroxide and iodine used concurrently against 3 bacterial and 16 yeast species.

**Results:**

Synergistic or additive inhibitory effects were shown for hydrogen peroxide and iodine mixtures against all 19 species used in the study. Both biocides were mostly cidal individually and in mixtures against *Pseudomonas aeruginosa* and *Staphylococcus aureus.* Both compounds manifested static inhibitory effects individually, but their mixtures were synergistically cidal for *Saccharomyces cerevisiae* and *Escherihia coli*. Cells of *S*. *cerevisiae* treated with hydrogen peroxide and iodine-hydrogen peroxide mixture produced increased numbers of respiratory deficient mutants indicating genotoxic effects.

**Conclusion:**

Iodine and hydrogen peroxide used concurrently interact synergistically or additively against a range of prokaryotic and eukaryotic microorganisms. The study provides an insight as to how these traditional antimicrobials could be used more effectively for disinfection and antisepsis. In addition, a simple approach is proposed for scoring genotoxicity of different biocides by using the budding yeast system.

## Background

Antimicrobials are extensively utilized for infection and microbial control in health care, industry and the environment [1]. They are also used for medical treatments [2]. Combining antimicrobials could enhance their activities (via additive effects or synergism) and could help to overcome acquired microbial resistance to single chemicals [1].

Iodine (I_2_) and hydrogen peroxide (H_2_O_2_) are oxidizing agents with a long history of usage as antimicrobials [1-6]. Iodine is a halogen releasing agent manifesting rapid bactericidal, fungicidal, virucidal and sporicidal effects caused by inhibiting DNA synthesis and attacking amino acids, nucleotides and fatty acids [1,7]. It is often used in complexes (iodophores) with solubilising agents [1,7]. Hydrogen peroxide is a peroxygen used for efficient control of various bacteria (especially Gram-positive), their spores, yeasts and viruses - due to the formation of free OH-radicals breaking DNA and oxidizing thiol-groups of proteins and lipids [1]. Both compounds are common antiseptics and disinfectants in topical skin therapy [4,8], wound healing [2,7,9-11], preparation of preoperative sites [12], control of gingival plaques [13], treating biofilms [14] and Fournier’s gangrene [15], disinfection of catheters [16] and other surfaces [17], industrial treatments of fish eggs [18,19], reducing bacterial pathogens on fruits [20], purification of water systems [21] and many other processes.

Some problems associated with side effects and acquired microbial resistance to single antimicrobials could be minimised by using them at lower concentrations in combination. Prerequisites for decreasing concentrations of biocides should be their synergistic or additive effects in mixtures. Increased antimicrobial activities of hydrogen peroxide were shown in combinations with other compounds including hypothiocyanite [22], sodium bicarbonate [23], rifampicin [24], neucoproine [25], chlorhexidine [26], different organic acids [20,27] as well as with UV-irradiation [28]. Synergies were reported also for iodine combined with an essential oil [29], hyaluronan [10], chlorhexidine gluconate [30], polyacrylonitrile [31].

A few sporadic studies on combining iodine and hydrogen peroxide have been reported [32-34], but the nature of interactions between these compounds and the potential use of this combination against various microorganisms were not studied. In this study, we compared inhibitory effects and modes of action of iodine and hydrogen peroxide used separately and in mixtures against 3 bacterial and 16 yeast species.

## Results and discussion

### Enhanced growth inhibition of 16 yeast species exposed to hydrogen peroxide and iodine concurrently

**Figure 1:**
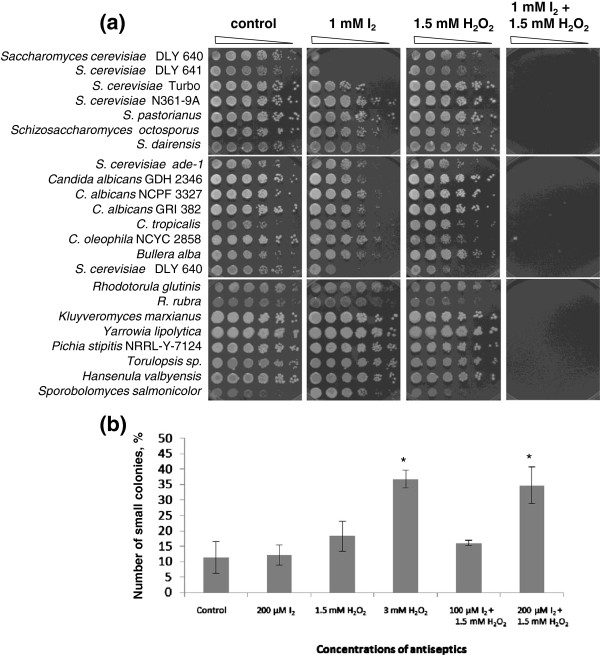
**Co-operative effects of hydrogen peroxide and iodine on yeast species. (a)** The enhancement in the inhibition of yeast growth by hydrogen peroxide and iodine used in the mixture. Six serial dilutions of each culture were plated in each horizontal row, with most concentrated suspensions on the left. **(b)** Enhanced incidence of small colonies in yeast cells treated with hydrogen peroxide and with the mixture iodine-hydrogen peroxide. Stars indicate significantly different values (P < 0.05).

In preliminary experiments, 2 mM iodine or 6 mM hydrogen peroxide (in solid YEPD medium) completely inhibited the growth of all tested *S*. *cerevisiae* haploid strains (data not shown). In spot tests carried out for 16 different yeast species (Figure 1; Additional file 1: Table S1) on solid YEPD medium containing 1 mM iodine and 1.5 mM hydrogen peroxide, haploid strains DLY 640 and DLY 641 (*S*. *cerevisiae*) were the most sensitive to iodine; strains Turbo, *ade*-*1* (*S*. *cerevisiae*), GDH 2346, NCPF 3327, GRI 382 (*C*. *albicans* ), species *C*. *tropicalis* and *Bullera alba* were slightly inhibited by this biocide; the remainder of the strains were not affected by iodine (Figure 1a). None of the strains was inhibited by 1.5 mM hydrogen peroxide. The mixture of both biocides completely inhibited the growth of all species.

These data show that all yeast species manifested cessation of their growth in the response to concurrent applications of iodine and hydrogen peroxide at concentrations allowing growth when the antiseptics were used separately. Therefore, it is very likely that this co-operative inhibitory capacity of the iodine-hydrogen peroxide mixture could be extended to many others, if not all, yeast species. Depending on the extent of microbial growth inhibition by any two combined substances, the interactions between them could be described as synergistic or additive [1,35]. To potentially extrapolate our results on prokaryotes, we investigated in more details the nature of inhibitory effects of iodine and hydrogen peroxide used separately and concurrently against three bacterial species, alongside with a budding yeast strain.

### Separate antimicrobial effects of hydrogen peroxide and iodine

We tested antimicrobial effects of hydrogen peroxide and iodine against budding yeast (*S*. *cerevisiae*) and three bacterial species (*Escherichia coli*, *Pseudomonas aeruginosa* and *Staphylococcus aureus*) grown in liquid culture.

MIC values of each compound varied for different species (Additional file 2: Table S2). *S*. *cerevisiae* and *S*. *aureus* were more sensitive to iodine than *E*. *coli* and *P*. *aeruginosa*. No visible growth of *S*. *cerevisiae* was detectable at 300 μM I_2_. 400 μM was a critical iodine concentration for *S*. *aureus*. The growth inhibition of *E*. *coli* and *P*. *aeruginosa* was observed only at 600 μM I_2._

*S*. *aureus* showed the highest sensitivity to H_2_O_2_. As no visible growth was detected at 200 μM H_2_O_2_, this concentration was taken as MIC. MIC of H_2_O_2_ for *E*. *coli* was 800 μM, for *S*. *cerevisiae* and *P*. *aeruginosa* it was 4 mM, which is 20 times higher than for *S*. *aureus* (Table S2).

In conclusion, various microorganisms manifested different sensitivities to iodine and hydrogen peroxide.

### Combined inhibitory effects of hydrogen peroxide and iodine

In mixtures of hydrogen peroxide and iodine, inhibitory concentrations were lower than for the individual compounds in liquid cultures of all tested species (Table 1). Criteria for synergism or additive effects are based on calculations of FIC and FICs values. FIC of a compound is the ratio of MIC value for this compound in the mixture to MIC value for this compound alone. The lower the FIC value is, the more effective inhibitory effect takes place in a mixture. The sum of FIC values (FICs = FIC_a_ + FIC_b_) determines interpreting the mode of inhibitory interactions between individual compounds (*a* and *b*) in a mixture [26,36]. For *E*. *coli*, at different combinations of hydrogen peroxide-iodine concentrations FICs indexes were in range 0.58-0.79 (less than 1) that could be interpreted as a strong additive interaction or a weak synergism [26,37]. Similar results were obtained for *P*. *aeruginosa* with FICs values between 0.58 and 0.75. For *S*. *cerevisiae* and *S*. *aureus* the effect of the interaction was additive, with FICs indexes around 1. Taking the definition of synergism at FICs values less than 0.5 [27,38] we could interpret our results as additive inhibitory effects.

**Table 1:** **Growth responses of *S *. *cerevisiae*, *E*. *coli*,*S*. ***aureus* and *P***.*aeruginosa *to I**_**2**_**and H**_**2**_**O**_**2**_**combined at various concentrations in liquid growth media**

***S*.*cerevisiae***							
I_2_ \H_2_O_2_*	0 μM	1 mM	1.5 mM	2 mM	2.5 mM	3 mM	4 mM
0 μM	+++	+++	+++	+++	+++	+++	-
20 μM	+++	+++	+++	+++	+++	+++	-
25 μM	+++	+++	+++	+++	+++	++	-
50 μM	+++	+++	+++	+++	+++	+	-
75 μM	+++	+++	+++	+++	+++	−^1^	-
100 μM	+++	+++	+++	+++	−^0.95^	-	-
150 μM	+++	+++	+++	+++	-	-	-
200 μM	+++	+++	++	−^1.16^	-	-	-
250 μM	+++	+++	++	−^1.33^	-	-	-
300 μM	-	-	-	-	-	-	-
***E*.*coli***							
I_2_ \H_2_O_2_	0 μM	100 μM	200 μM	400 μM	600 μM	800 μM	1 mM
0 μM	+++	+++	+++	+++	+++	-	-
50 μM	+++	+++	+++	−^0.58^	-	-	-
100 μM	+++	+++	+++	-	-	-	-
200 μM	+++	+++	++	-	-	-	-
300 μM	+++	+++	−^0.75^	-	-	-	-
400 μM	+++	−^0.79^	-	-	-	-	-
500 μM	-	-	-	-	-	-	-
***S*.*aureus***							
I_2_ \H_2_O_2_	0 μM	75 μM	100 μM	150 μM	200 μM	300 μM	400 μM
0 μM	+++	+++	+++	+++	-	-	-
100 μM	+++	+++	+++	−^1^	-	-	-
150 μM	+++	+++	+++	-	-	-	-
200 μM	+++	++	−^1^	-	-	-	-
250 μM	+++	−^1^	-	-	-	-	-
300 μM	+++	-	-	-	-	-	-
400 μM	-	-	-	-	-	-	-
***P*.*aeruginosa***							
I_2_ \H_2_O_2_	0 mM	1 mM	1.5 mM	2 mM	2.5 mM	3 mM	4 mM
0 μM	+++	+++	+++	+++	+++	+	-
25 μM	+++	+++	+++	+	−^0.66^	-	-
50 μM	+++	+++	+++	−^0.58^	-	-	-
100 μM	+++	+++	+	-	-	-	-
200 μM	+++	+++	−^0.71^	-	-	-	-
250 μM	+++	++	-	-	-	-	-
300 μM	+++	−^0.75^	-	-	-	-	-
600 μM	-	-	-	-	-	-	-

*Concentrations for H_2_O_2_ are shown in horizontal rows; concentrations for I_2_ are shown in vertical rows. The absence of growth is indicated by the sign “-“. FICs index values are shown as numbers near correspondent “-“ sign. Signs “+++”, “++” and “+” indicate different degrees of growth. The results were recorded after 2 days of cultivation at 30°C (for *S*. *cerevisiae*), or after 1–2 days of cultivation at 37°C (for *E*. *coli*, *S*. *aureus* and *P*. *aeruginosa*).

### Recovery of inhibited cells

To test modes of inhibition (cell death or growth arrest) caused by hydrogen peroxide and iodine, cells inhibited by treatments were washed twice, spread on plates without antimicrobials, and incubated under conditions permissive for growth. Different species manifested diverse abilities to recover the growth after inhibition by individual and mixed compounds.

Cells of *P*. *aeruginosa* treated with the individual compounds at MICs (600 μM for iodine and 4 mM for hydrogen peroxide) did not recover (Additional file 3: Figure S1). No recovery of *P*. *aeruginosa* was also found after combined treatments, even at concentrations lower than MIC values for each compound. For example, the mixture (200 μM I_2_ + 1.5 mM H_2_O_2_), where concentration of iodine was 3 times lower and concentration of H_2_O_2_ was 2.7 times lower than for individual MICs, killed cells of *P*. *aeruginosa* irreversibly.

There were no viable cells after treatment of *S*. *aureus* with 400 μM iodine (MIC), but substantial amounts of cells were viable after treatment with 250 μM hydrogen peroxide (MIC for H_2_O_2_ is 200 μM). Small amounts of CFU were found in 2 out of 3 tested combinations of both antimicrobials, (250 μM I_2_ + 75 μM H_2_O_2_) and (100 μM I_2_ + 150 μM H_2_O_2_), with 33 colonies and 32 colonies respectively (both from undiluted cultures). No colonies appeared from the mixture (300 μM I_2_ + 150 μM H_2_O_2_). We concluded that for *S*. *aureus* iodine is more cidal than hydrogen peroxide (Additional file 3: Figure S2).

The above data suggest that cells of *P*. *aeruginosa* and *S*. *aureus* were killed more effectively by iodine and hydrogen peroxide used in mixtures rather than individually, and that inhibitory effects were achieved at lower concentrations of the antiseptics due to synergism.

Cells of *S*. *cerevisiae* treated either with iodine or hydrogen peroxide recovered after applications of these compounds at all tested concentrations. No recovery was observed after combined treatments at lower concentrations (Additional file 3: Figure S3). Similarly, no recoveries of cell divisions were observed after combined treatments of *E*. *coli* with two antiseptics (cidal effects), while recoveries took place after individual treatments with either iodine or hydrogen peroxide (Additional file 3: Figure S4). These data indicate that at least some proportions of treated cells of *S*. *cerevisiae* and *E*. *coli* were not able to divide due to static effects. The combined treatments of these species resulted in complete loss of their viabilities, implying that synergism led to cidal action.

Cidal effects are more advantageous than static ones, in terms of effectiveness. Therefore, combined treatments with iodine-hydrogen peroxide could be more effective approach in many aspects of disinfection and topical treatments associated with these antiseptics. Particular advantages of the combined treatments could be related to reduced concentrations of the antimicrobials that could be important for reducing their side effects [39,40]. Another advantage would be prevention of microbial resistance that could be acquired to single biocides [41,42].

Our experiments were carried out *in vitro* on pure cultures of microorganisms. Application of the co-operative inhibitory effects to *in vivo* clinical conditions including potentially mixed infections and biological substrates will require additional trials. However, a recent clinical study demonstrated effective reductions of post-operational infections by using combined treatments with PVP-iodine and hydrogen peroxide after spine surgeries [33].

### Iodine enhances incidence of respiration deficiencies in yeast cells treated with hydrogen peroxide

Treatments of budding yeast cells with hydrogen peroxide and mixtures hydrogen peroxide-iodine (followed by washing with water) increased numbers of small colonies on solid YEPD. Their largest numbers (36.8% of total colony number) were produced by cells treated with hydrogen peroxide at concentration 3 mM (Figure 1b). After treatment with 1.5 mM hydrogen peroxide the slight increase in the number of small colonies (18% as average) was not statistically significant as compared with control (11% of small colonies). Treatments with up to 200 mM iodine did not increase numbers of small colonies (12%). However, cells treated with the mixture of hydrogen peroxide-iodine (1.5 mM + 200 μM respectively) produced 34% of small colonies suggesting a co-operative effect between hydrogen peroxide and iodine. Under these treatment conditions cells were clearly inhibited but were still able to grow.

We reasoned that small colonies might present the *petite* mutants associated with mutations in mitochondrial DNA leading to respiratory deficiencies. The *petite* mutants are not able to grow under respiration conditions, for example, on glycerol or ethanol [43]. Indeed, most of randomly selected individual small colonies from treatments with 3 mM hydrogen peroxide (78%) and with the mixture 1.5 mM hydrogen peroxide + 200 μM iodine (79%) were not able to grow on YEPG medium and, therefore, were respiratory deficient (data not shown).

The increased incidence of *petite* mutants indicates that the antimicrobials produce genotoxic effects. In case of hydrogen peroxide they could be associated with oxidative stress via intensive generation of reactive oxygen species that enhance mutation rates [44]. Iodine itself did not affect spontaneous frequencies of small colonies. However, combined with hydrogen peroxide, iodine enhanced the effect of this compound (Figure 1b) suggesting some additional (possibly, co-operative) contribution to the genotoxic effect of hydrogen peroxide. Since the respiratory deficient yeast mutants are easily detectable, this approach could be potentially used for scoring genotoxic effects of any other antimicrobials (including new ones) - based on numbers of small colonies appearing after treatments. The enhanced genotoxicity itself could contribute to stronger biocidal effects against microorganisms. On the other hand, genotoxicity might increase chances for microbial mutations of drug resistance. However, the biocidal activity of the combined antimicrobials at appropriate concentrations would prevent the survival of mutants. The other potential issue is genotoxicity of antimicrobials for human cells. Overall, interplay between these processes could be the subject of further studies.

## Conclusions

Synergistic and additive inhibitory effects of hydrogen peroxide and iodine shown for 3 bacterial and 16 yeast species imply possibilities of more effective concurrent usage of these traditional antimicrobials in various applications. Combining these compounds often converts their individual static inhibitory effects into cidal effects. Synergism is also manifested in higher frequencies of respiratory deficient yeast cells suggesting enhanced genotoxicity of the mixed antimicrobials.

## Methods

### Strains and culture conditions

3 bacterial and 16 yeast species were used (Additional file 1: Table S1). Bacteria were cultivated on nutrient agar or in nutrient broth for one day at 37°C. Yeasts were cultivated on solid or liquid YEPD (yeast extract, peptone, dextrose) medium at 30°C for 2 days or at 23°C for 3 days. Fresh yeast cultures were used to inoculate liquid YEPD medium at densities about 5×10^5^ cells/ml. Single colonies of bacterial strains cultivated on nutrient agar at 37°C for 16–18 hours were used for inoculating nutrient broth at densities of approximately 5×10^5^ cells/ml.

### Spot tests

Fresh yeast cells grown in YEPD were resuspended in sterile distilled water to OD ~ 0.5 at 660 nm. 5 fold serial dilutions of each strain in 96-well plates (200 μl each) were plated in two replicas with Sigma-Aldrich plater onto YEPD plates with iodine and/or hydrogen peroxide, or without them. Plates were incubated at 23°C for 3 days.

### Determination of minimum inhibitory concentration (MIC)

MIC values were determined separately for hydrogen peroxide and iodine (Sigma). The aqueous stock solution of iodine (product number 318981) contained KI as a solubility stabiliser. 2 ml of cultures (5×10^5^ cells/ml) were added to test tubes, and different amounts from serial dilutions of 1 M H_2_O_2_ or 50 mM I_2_ stock solutions were added to the cultures to obtain a range of concentrations (10 μM - 10 mM for H_2_O_2_, and 10 μM - 1 mM for I_2_). Cultures were incubated at 30°C for 2 days (yeast) and at 37°C for 1–2 days (bacteria) to determine MICs (the lowest concentrations preventing growth).

### Determination of the interaction mode between hydrogen peroxide and iodine

To determine MICs for individual mixtures of both compounds, serial dilutions of H_2_O_2_ and I_2_ were combined in test tubes containing 2 ml of cultures (~5×10^5^ cells/ml). Cultures were incubated at 30°C for 2 days (yeast) or at 37°C for 1–2 days (bacteria). MIC values for each H_2_O_2_-I_2_ mixture were determined as described above. Fractional inhibitory concentration (FIC) of each compound was calculated according to the formula: FIC = MIC of a compound in a mix/MIC of a compound alone [26,36]. The sum of fractional inhibitory concentrations (FICs) was calculated as follows: FICs = FIC_*hydrogen peroxide*_ + FIC_*iodine*_ FICs indices less than 1 were interpreted as weak synergistic interactions or strong additive effects.

### Tests for growth recovery after inhibition with biocides

To test if growth inhibition was caused by cell death or by static inhibition of cell divisions, cells were washed from biocides 2 times with nutrient broth (for bacteria) or sterile distilled water (for yeast), and their serial dilutions were plated on freshly poured solid media for counting colonies after 1 and 2 days of growth at 30°C (yeast) and 37°C (bacteria).

### Testing respiratory status of *S*. *cerevisiae* clones

Water suspensions of randomly selected individual small colonies were patched onto two types of solid media: YEPD (containing glucose, 2%) and YEPG (containing glycerol, 3%). Plates were incubated for 2.5-3 days at 30°C. Cells growing only on YEPD (fermentation conditions) but not growing on YEPG (respiration conditions) were classified as respiratory deficient mutants; cells growing on both media were considered as respiratory proficient [43].

### Statistical analysis

Quantitative data were presented as means and standard deviations (SD). Differences with P values less than 0.05 determined in t-tests were considered as significant.

## Competing interests

The authors declare that they have no competing interests.

## Authors’ contributions

EIZ and MKZ designed the study. EIZ has performed predominant part of experiments. MKZ did spot tests and wrote the bulk of the manuscript. EIZ calculated data and made tables and figures. Both authors approved the final version of the manuscript.

## Supplementary Material

Additional file 1: Table S1 Microorganisms used in the study. [45,46]. Click here for file

Additional file 2: Table S2 Growth responses of budding yeast (*S*. *cerevisiae*) and three bacterial species (*S*.*aureus*, *P*. *aeruginosa* and *E*.*coli*) to different concentrations of H_2_O_2_ and I_2_ in culture media. Click here for file

Additional file 3: Figure S1 Growth responses of *P*. *aeruginosa* to different concentrations of I_2_**(a)**, H_2_O_2_**(b)** and mixtures of I_2_ and H_2_O_2_ at different concentrations in nutrient broth **(c)**. **(d)** Recovery of the growth after inhibition with individual and mixed compounds. 20 μl from undiluted cultures and from two different dilutions (100 fold and 1000 fold) were plated on each plate and grown overnight at 37°C, with further checking on the next day. **Figure S2.** Growth responses (growth or no growth) of *S*. *aureus* to different concentrations of I_2_**(a)**, H_2_O_2_**(b)** and mixtures of I_2_ and H_2_O_2_ at different concentrations in nutrient broth **(c)**. **(d)** Recovery of the growth after inhibition with individual and mixed compounds. 20 μl from undiluted cultures and from two different dilutions (100 fold and 1000 fold) were plated on each plate and grown overnight at 37°C, with further checking on the next day. **Figure S3.** Growth responses (growth or no growth) of *S*. *cerevisiae* to different concentrations of I_2_**(a)**, H_2_O_2_**(b)** and mixtures of I_2_ and H_2_O_2_ at different concentrations in YEPD liquid medium **(c)**. **(d)** Recovery of the growth after inhibition with individual and mixed compounds. Before plating cultures were diluted 1000 times and 100 times. In cases of mixed compounds, both diluted and undiluted cultures were plated. The plated were incubated for 2 days at 30°C. **Figure S4.** Growth responses (growth or no growth) of *E*. *coli* to different concentrations of I_2_**(a)**, H_2_O_2_**(b)** and mixtures of I_2_ and H_2_O_2_ at different concentrations in nutrient broth **(c)**. **(d)** Recovery of the growth after inhibition with individual and mixed compounds. 20 μl from undiluted cultures and from two different dilutions (100 fold and 1000 fold) were plated on each plate and grown overnight at 37°C, with further checking on the next day. Click here for file
