# Supplementary material for: Co-operative inhibitory effects of hydrogen peroxide and iodine against bacterial and yeast species
Source: BMC Res Notes. 2013 Jul 15;6:272. doi: 10.1186/1756-0500-6-272 (PMC3716994; doi:10.1186/1756-0500-6-272)
Supplement: Additional file 1: Table S1 — Microorganisms used in the study. [45,46]. [file 1756-0500-6-272-S1.docx]

**Table S1:** Microorganisms used in the study.

**Species Strain information**

**Bacteria**

*Escherichia coli*  NCTC 9001

*Pseudomonas aeruginosa*  NCTC 6749

*Staphylococcus aureus* NCTC 6571

**Yeasts**

*Bullera alba* MMU collection

*Candida albicans* GDH 2346 (MMU collection)

*C. albicans* GRI 382 (MMU collection)

*C. albicans*  NCPF 3327 [45]

*C. oleophila* NCYC 2858 (MMU collection)

*C. tropicalis* MMU collection

*Hansenula valbyensis* (MMU collection)

*Kluyveromyces marxianus* (MMU collection)

*Pichia* *stipitis* NRRL-Y-7124

*Rhodotorula glutinis* (MMU collection)

*R. rubra* (MMU collection)

*Saccharomyces cerevisiae ade-1* mutant (MMU collection)

*S. cerevisiae*  DLY 640 [46]

*S. cerevisiae*  DLY 641 [46]

*S. cerevisiae*  N361-9A (MMU collection)

*S. cerevisiae*  Turbo (MMU collection)

*S. dairensis* (MMU collection)

*S. pastorianus* (MMU collection)

*Schizosaccharomyces octosporus* (MMU collection)

*Sporobolomyces salmonicolor* (MMU collection)

*Torulopsis sp.* (unknown species) (MMU collection)

*Yarrowia lipolytica* (MMU collection)
