# Supplementary material for: Co-operative inhibitory effects of hydrogen peroxide and iodine against bacterial and yeast species
Source: BMC Res Notes. 2013 Jul 15;6:272. doi: 10.1186/1756-0500-6-272 (PMC3716994; doi:10.1186/1756-0500-6-272)
Supplement: Additional file 2: Table S2 — Growth responses of budding yeast (S. cerevisiae) and three bacterial species (S.aureus, P. aeruginosa and E.coli) to different concentrations of H2O2 and I2 in culture media. [file 1756-0500-6-272-S2.docx]

**Table S2:** Growth responses of budding yeast (*S. cerevisiae*) and three bacterial species (*S.aureus*, *P. aeruginosa* and *E.coli*) to different concentrations of H_2_O_2_ and I_2_ in culture media.

| **H_2_O_2_** concentration | Growth (+/-) | | | |
| --- | --- | --- | --- | --- |
|  | *S. aureus* | *P. aeruginosa* | *E. coli* | *S. cerevisiae* |
| 10 µM | **+** | **+** | **+** | **+** |
| 25 µM | **+** | **+** | **+** | **+** |
| 50 µM | **+** | **+** | **+** | **+** |
| 75 µM | **+** | **+** | **+** | **+** |
| 100 µM | **+** | **+** | **+** | **+** |
| 150 µM | **+** | **+** | **+** | **+** |
| 200 µM | **-** | **+** | **+** | **+** |
| 250 µM | **-** | **+** | **+** | **+** |
| 300 µM | **-** | **+** | **+** | **+** |
| 400 µM | **-** | **+** | **+** | **+** |
| 500 µe | **-** | **+** | **+** | **+** |
| 600 µM | **-** | **+** | **+** | **+** |
| 800 µM | **-** | **+** | **-** | **+** |
| 1mM | **-** | **+** | **-** | **+** |
| 1.5mM | **-** | **+** | **-** | **+** |
| 2mM | **-** | **+** | **-** | **+** |
| 2.5mM | **-** | **+** | **-** | **+** |
| 3mM | **-** | **+** | **-** | **+** |
| 4mM | **-** | **­-** | **-** | **-** |
| 5mM | **-** | **-** | **-** | **-** |
| 10mM | **-** | **-** | **-** | **-** |

| **I_2_** concentration | Growth +/- | | | |
| --- | --- | --- | --- | --- |
|  | *S. aureus* | *P.*  *aeruginosa* | *E.coli* | *S. cerevisiae* |
| 25 µM | **+** | **+** | **+** | **+** |
| 50 µM | **+** | **+** | **+** | **+** |
| 100 µM | **+** | **+** | **+** | **+** |
| 150 µM | **+** | **+** | **+** | **+** |
| 200 µM | **+** | **+** | **+** | **+** |
| 250 µM | **+** | **+** | **+** | **+** |
| 300 µM | **+** | **+** | **+** | **-** |
| 400 µM | **-** | **+** | **+** | **-** |
| 500 µM | **-** | **+** | **+** | **-** |
| 600 µM | **-** | **­-** | **-** | **-** |
| 700 µM | **-** | **-** | **-** | **-** |
| 800 µM | **-** | **-** | **-** | **-** |
| 900 µM | **-** | **-** | **-** | **-** |
| 1mM | **-** | **-** | **-** | **-** |

For each compound, the lowest concentration at which visually detectable growth (turbidity) did not take place was taken as a minimum inhibitory concentration (MIC)_._ MIC values are shown as shaded boxes.
