# Supplementary material for: Co-operative inhibitory effects of hydrogen peroxide and iodine against bacterial and yeast species
Source: BMC Res Notes. 2013 Jul 15;6:272. doi: 10.1186/1756-0500-6-272 (PMC3716994; doi:10.1186/1756-0500-6-272)
Supplement: Additional file 3: Figure S1 — Growth responses of P. aeruginosa to different concentrations of I2(a), H2O2(b) and mixtures of I2 and H2O2 at different concentrations in nutrient broth (c). (d) Recovery of the growth after inhibition with individual and mixed compounds. 20 μl from undiluted cultures and from two different dilutions (100 fold and 1000 fold) were plated on each plate and grown overnight at 37°C, with further checking on the next day. Figure S2. Growth responses (growth or no growth) of S. aureus to different concentrations of I2(a), H2O2(b) and mixtures of I2 and H2O2 at different concentrations in nutrient broth (c). (d) Recovery of the growth after inhibition with individual and mixed compounds. 20 μl from undiluted cultures and from two different dilutions (100 fold and 1000 fold) were plated on each plate and grown overnight at 37°C, with further checking on the next day. Figure S3. Growth responses (growth or no growth) of S. cerevisiae to different concentrations of I2(a), H2O2(b) and mixtures of I2 and H2O2 at different concentrations in YEPD liquid medium (c). (d) Recovery of the growth after inhibition with individual and mixed compounds. Before plating cultures were diluted 1000 times and 100 times. In cases of mixed compounds, both diluted and undiluted cultures were plated. The plated were incubated for 2 days at 30°C. Figure S4. Growth responses (growth or no growth) of E. coli to different concentrations of I2(a), H2O2(b) and mixtures of I2 and H2O2 at different concentrations in nutrient broth (c). (d) Recovery of the growth after inhibition with individual and mixed compounds. 20 μl from undiluted cultures and from two different dilutions (100 fold and 1000 fold) were plated on each plate and grown overnight at 37°C, with further checking on the next day. [file 1756-0500-6-272-S3.ppt]

## Slide 1
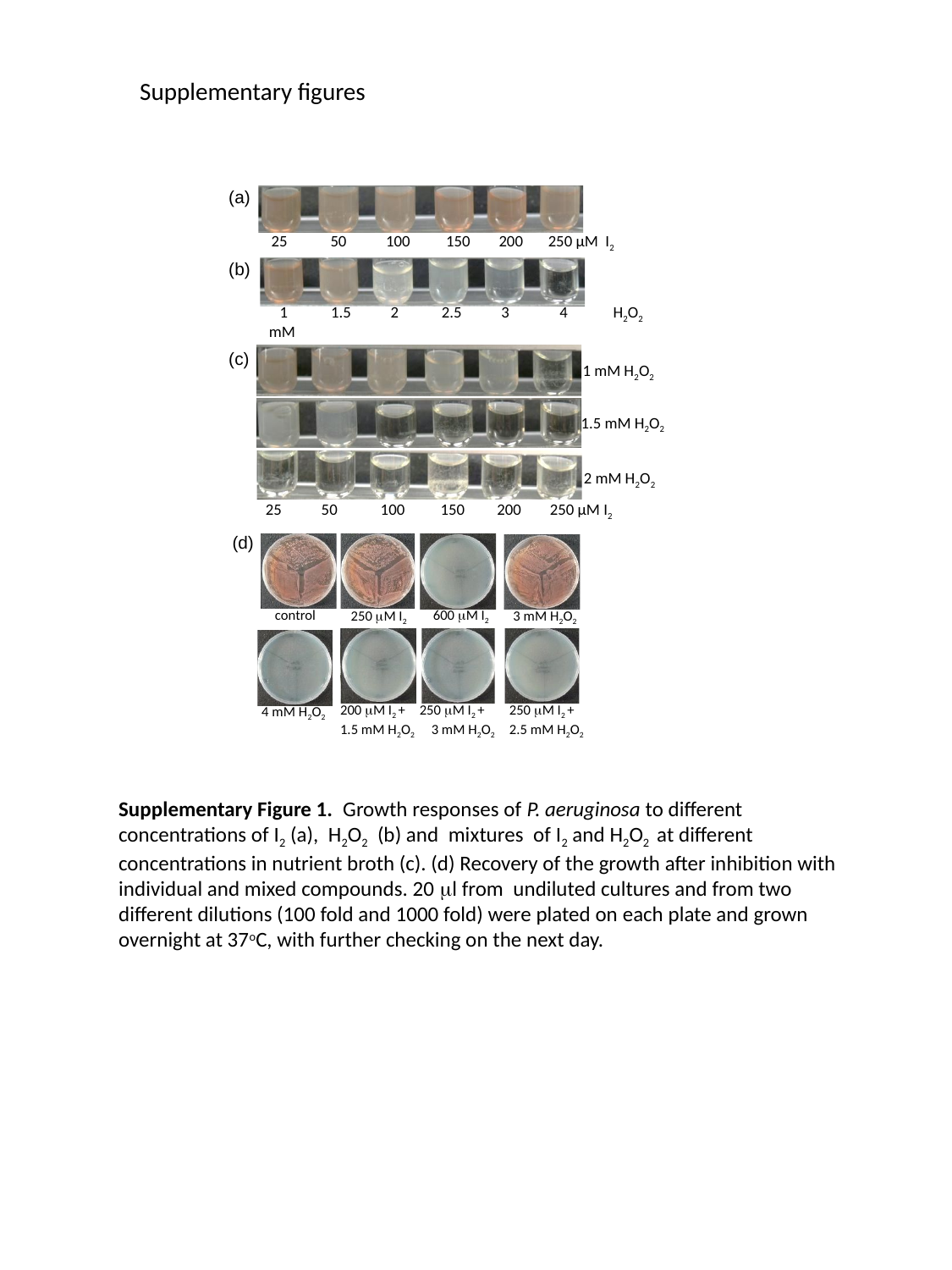

Supplementary figures
(a)
 25 50 100 150 200 250 µM I2
(b)
H2O2
 1 1.5 2 2.5 3 4 mM
(c)
1 mM H2O2
1.5 mM H2O2
2 mM H2O2
 25 50 100 150 200 250 µM I2
(d)
control
250 μM I2
600 μM I2
3 mM H2O2
 200 μM I2 + 1.5 mM H2O2
250 μM I2 + 3 mM H2O2
250 μM I2 + 2.5 mM H2O2
4 mM H2O2
Supplementary Figure 1. Growth responses of P. aeruginosa to different concentrations of I2 (a), H2O2 (b) and mixtures of I2 and H2O2 at different concentrations in nutrient broth (c). (d) Recovery of the growth after inhibition with individual and mixed compounds. 20 μl from undiluted cultures and from two different dilutions (100 fold and 1000 fold) were plated on each plate and grown overnight at 37oC, with further checking on the next day.

## Slide 2
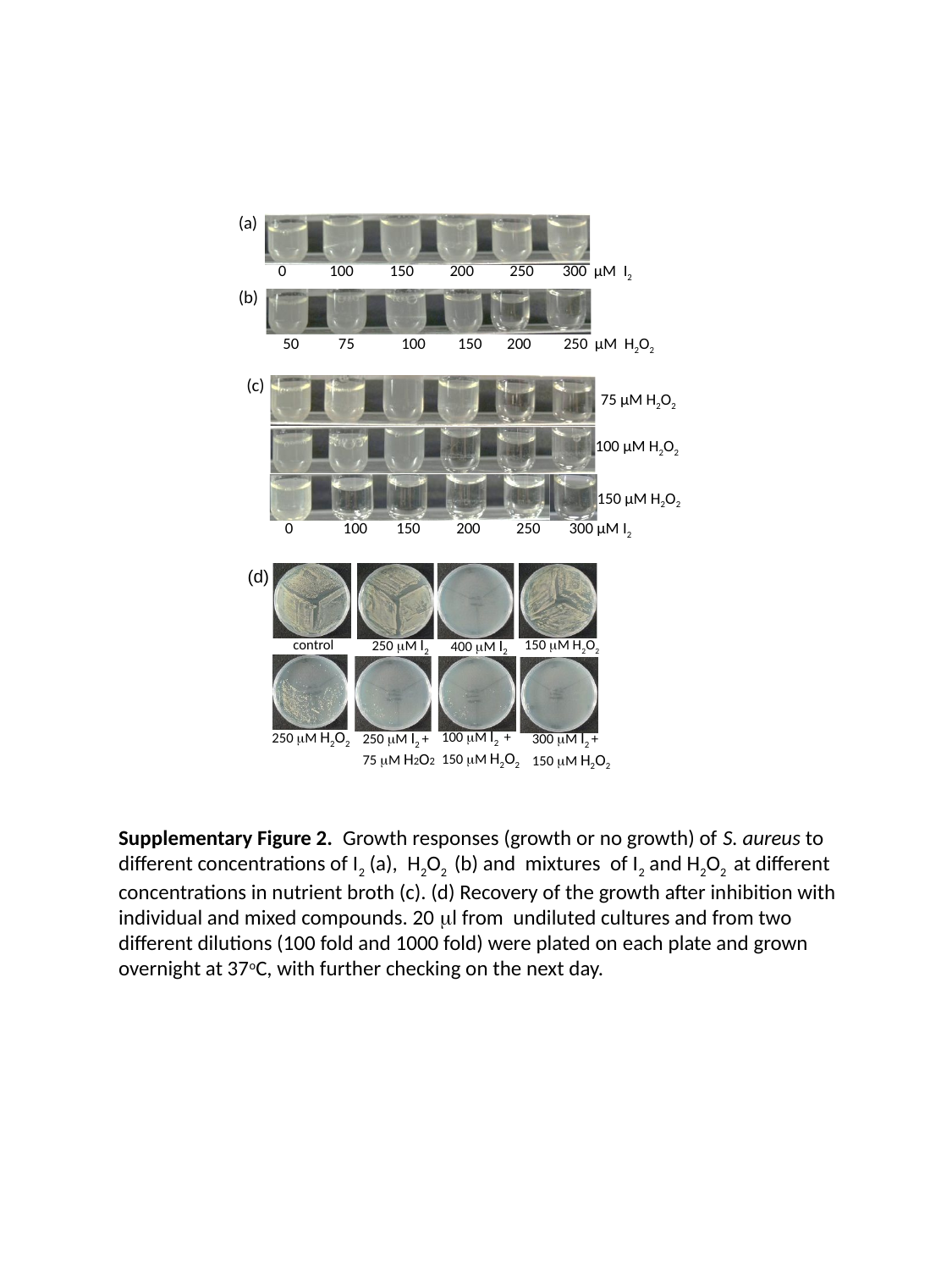

(a)
 0 100 150 200 250 300 µM I2
(b)
 50 75 100 150 200 250 µM H2O2
(c)
75 µM H2O2
100 µM H2O2
150 µM H2O2
 0 100 150 200 250 300 µM I2
(d)
150 μM H2O2
control
250 μM I2
400 μM I2
250 μM H2O2
100 μM I2 + 150 μM H2O2
250 μM I2 + 75 μM H2O2
300 μM I2 + 150 μM H2O2
Supplementary Figure 2. Growth responses (growth or no growth) of S. aureus to different concentrations of I2 (a), H2O2 (b) and mixtures of I2 and H2O2 at different concentrations in nutrient broth (c). (d) Recovery of the growth after inhibition with individual and mixed compounds. 20 μl from undiluted cultures and from two different dilutions (100 fold and 1000 fold) were plated on each plate and grown overnight at 37oC, with further checking on the next day.

## Slide 3
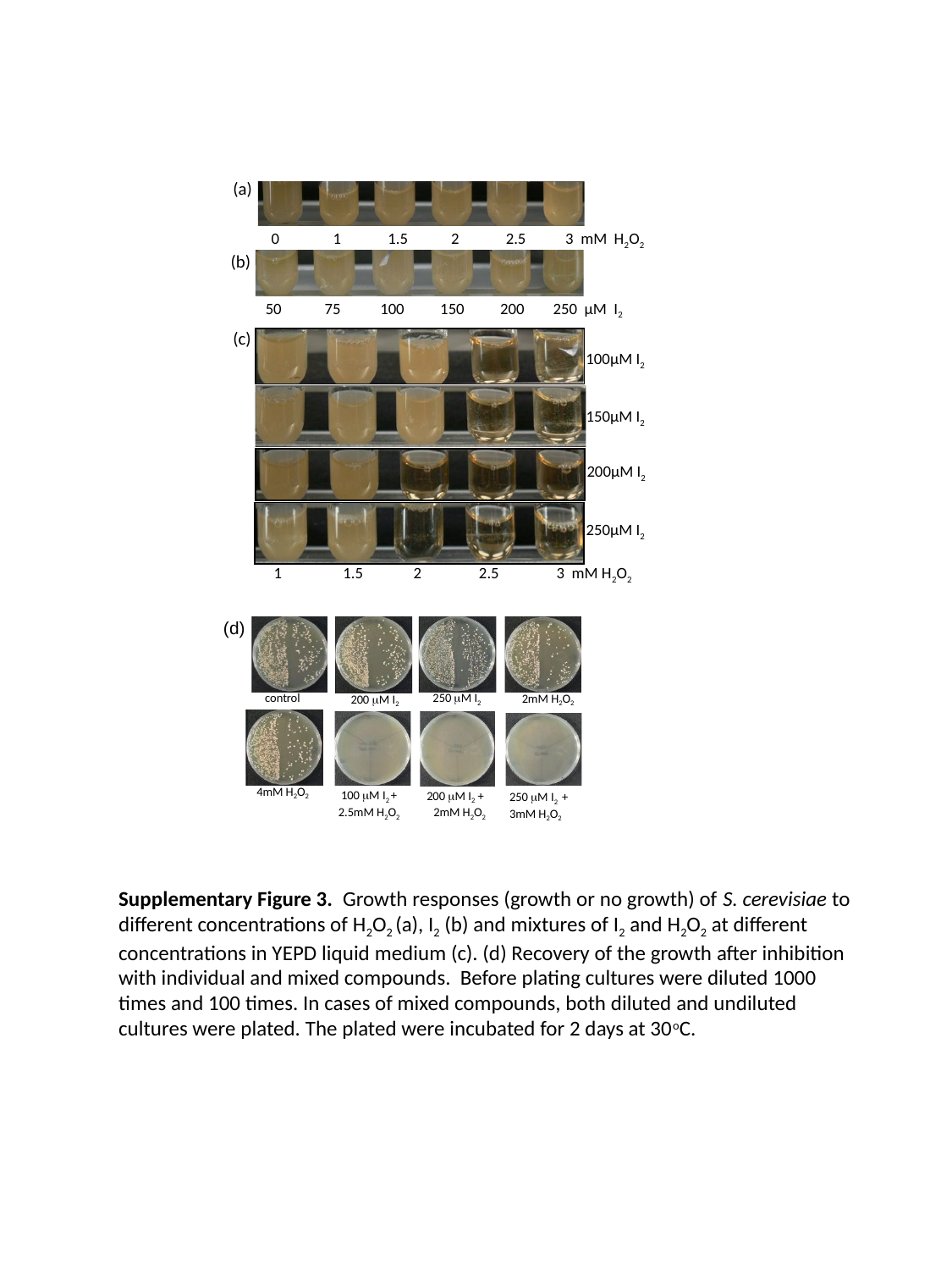

(a)
(a)
 0 1 1.5 2 2.5 3 mM H2O2
 (b)
 50 75 100 150 200 250 µM I2
(b)
(c)
 c)
100µM I2
150µM I2
 200µM I2
250µM I2
 1 1.5 2 2.5 3 mM H2O2
(d)
control
200 μM I2
250 μM I2
2mM H2O2
4mM H2O2
100 μM I2 + 2.5mM H2O2
200 μM I2 + 2mM H2O2
250 μM I2 + 3mM H2O2
Supplementary Figure 3. Growth responses (growth or no growth) of S. cerevisiae to different concentrations of H2O2 (a), I2 (b) and mixtures of I2 and H2O2 at different concentrations in YEPD liquid medium (c). (d) Recovery of the growth after inhibition with individual and mixed compounds. Before plating cultures were diluted 1000 times and 100 times. In cases of mixed compounds, both diluted and undiluted cultures were plated. The plated were incubated for 2 days at 30oC.

## Slide 4
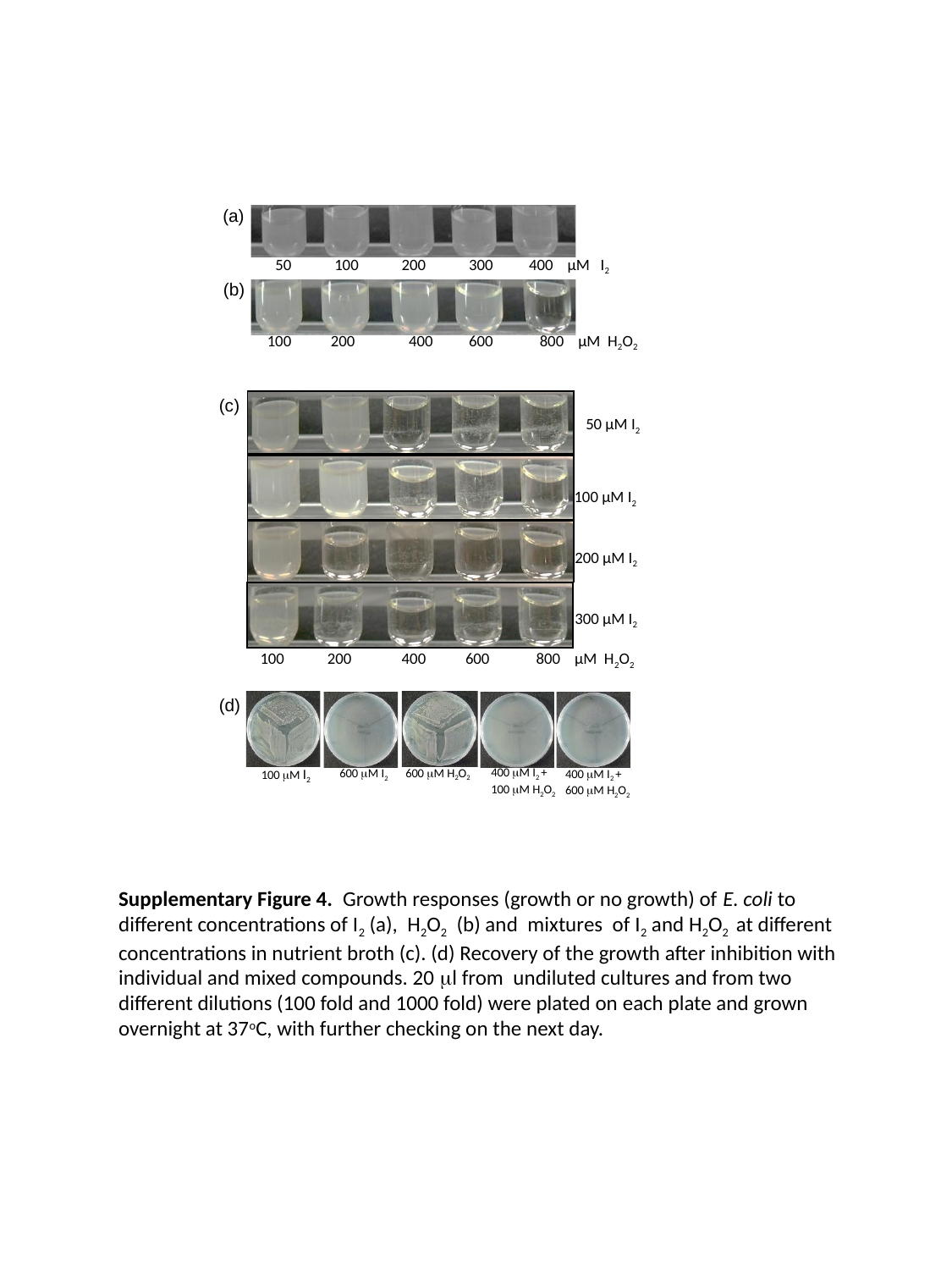

(a)
50 100 200 300 400 µM I2
(b)
 100 200 400 600 800 µM H2O2
(c)
50 µM I2
100 µM I2
 200 µM I2
 300 µM I2
 100 200 400 600 800 µM H2O2
(d)
100 μM I2
600 μM H2O2
600 μM I2
400 μM I2 + 100 μM H2O2
400 μM I2 + 600 μM H2O2
Supplementary Figure 4. Growth responses (growth or no growth) of E. coli to different concentrations of I2 (a), H2O2 (b) and mixtures of I2 and H2O2 at different concentrations in nutrient broth (c). (d) Recovery of the growth after inhibition with individual and mixed compounds. 20 μl from undiluted cultures and from two different dilutions (100 fold and 1000 fold) were plated on each plate and grown overnight at 37oC, with further checking on the next day.
